# Development of a multidisciplinary medication management program in nursing homes: protocol for a randomized controlled trial: Multidisciplinary medication management in nursing homes

**DOI:** 10.1186/s12877-024-04844-2

**Published:** 2024-03-04

**Authors:** Hye Jun Lee, Sunmee Jang, Ju-Yeun Lee, Young-Mi Ah, Mi-Kyung Lee, Suhyun Jang, Sena An, Jung-Ha Kim

**Affiliations:** 1 Department of Family Medicine, Chung-Ang University Hospital, College of Medicine, Chung-Ang University, Seoul, 06973 South Korea; 2 College of Pharmacy and Gachon Institute of Pharmaceutical Sciences, Gachon University, Incheon, South Korea; 3 College of Pharmacy and Research Institute of Pharmaceutical Sciences, Seoul National University, Seoul, South Korea; 4 College of Pharmacy, Yeungnam University, Gyeongsan, Gyeongbuk South Korea; 5 Department of Laboratory Medicine, Chung-Ang University Hospital, College of Medicine, Chung-Ang University, Seoul, South Korea

**Keywords:** Polypharmacy, Multidisciplinary medication management, Comprehensive medication review, Deprescribing, Long-term care facility residents

## Abstract

**Background:**

Polypharmacy and the use of potentially inappropriate medications are common among nursing home residents and are associated with negative outcomes. Although deprescribing has been proposed as a way to curtail these problems, the best way to implement multidisciplinary comprehensive medication review and deprescribing and its real impact in specific high-risk populations, such as nursing home residents, is still unclear. This multicenter randomized controlled clinical trial aims to assess the effects of a multidisciplinary mediation management program on medication use and health problems.

**Methods:**

A total of 1,672 residents aged ≥ 65 years from 22 nursing homes in South Korea who meet the targeted criteria, such as the use of ≥ 10 medications, are eligible to participate. The experimental group will receive a comprehensive medication review, deprescription, and multidisciplinary case conference with the help of platform. Outcomes will be measured at baseline, at the end of the intervention, as well as at 3, 6, 9, and 12 months after the end of the intervention. The primary endpoints will be the rate of adverse drug events, number of potentially inappropriate medications/potentially inappropriate medication users/two or more central nervous system drug/ central nervous system drug users, delirium, emergency department visits, hospitalization, and falls. The secondary endpoint will be the number of medications taken and polypharmacy users.

**Discussion:**

Our trial design is unique in that it aims to introduce a structured operationalized clinical program focused on reducing polypharmacy and potentially inappropriate medications in a nursing home setting with large samples.

**Trial registration:**

Ethical approval was granted by the public institutional review board of the Ministry of Health and Welfare (2022-1092-009). The study is also registered with the Clinical Research Information Service (Identifier: KCT0008157, Development and evaluation of a multidisciplinary medication management program in long-term care facility residents Status: Approved First Submitted Date: 2023/01/18 Registered Date: 2023/02/03 Last Updated Date: 2023/01/18 (nih.go.kr) https://cris.nih.go.kr/), which includes all items from the World Health Organization Trial Registration Dataset.

**Supplementary Information:**

The online version contains supplementary material available at 10.1186/s12877-024-04844-2.

## Background

A high prevalence of polypharmacy, defined as the concurrent use of five or more drugs, is observed among older adults [1]. Although sometimes necessary to treat multiple conditions, polypharmacy has many drawbacks, the most important being an increased risk of drug-related problems, and is a heavy burden for both the patient and the healthcare system [2]. In addition, polypharmacy increases the probability of the use of potentially inappropriate medications (PIMs) [2]. Older adults living in long-term care facilities (LTCFs) are at risk of polypharmacy and PIM use. One study reported that 70% of LTCF residents received five or more prescribed medications [3], and another study reported that approximately 40% of LTCF residents received PIMs compared to 20–25% of older adults living in the community [4]. In South Korea, the average number of medications taken by residents in an LTCF was reportedly 10.6 ± 4.2, those taking ≥ 11 constituted 45.7% [5], and PIM users were reported at 40.7% [6].

Recently, deprescribing, the process of tapering or stopping drugs supervised by a multidisciplinary healthcare professional aimed at minimizing polypharmacy and improving patient outcomes, has emerged as a useful way to reduce both polypharmacy and PIM use [7, 8]. However, research conducted in LTCF residents is still scarce. Furthermore, no systematic approaches to practice are routinely embedded in clinical care to reduce polypharmacy and PIM use among LTCF residents [9].

In this study, we propose testing a multidisciplinary medication management program (MMMP) for LTCF residents, an intervention designed to be suitable for routine use in LTCF settings. We hypothesized that deprescribing via the MMMP would decrease drug-related problems, including the number of medications and PIMs, and improve health-related outcomes.

## Methods/design

### Aim

We will assess the effects of implementing the MMMP in addressing polypharmacy and PIM use and on a range of health-related outcomes and healthcare service use in adults aged ≥ 65 years who meet the targeted criteria in an LTCF setting. The study will be conducted using a cluster randomized controlled trial (RCT) design with participants belonging to the same group (intervention or control group) within the LTCF.

### Multidisciplinary medication management program in long-term care facility residents

The MMMP in LTCF residents is a structured operationalized clinical pathway for comprehensive medication review (CMR), deprescribing, and multidisciplinary case conferences (MCC) by a team that includes visiting physicians, pharmacists, and nurses in partnership with the participants. In South Korea’s LTCF, a physician is not a resident, but they work outside and regularly visit the LTCF to administer treatment. Therefore, LTCF residents receive treatment from these physicians (visiting physicians) or from external physicians who treated them before they entered the LTCF. The MMMP uses an underlying secure digital platform (pacen-mmmpin.com (PACEN (website.ne.kr)], which integrates evidence to support multiple tools to check whether it is PIMs or not and cumulative medication burdens. This platform integrates consultation elements and evidence support across providers in shared electronic records.

### Design

**Fig. 1:**
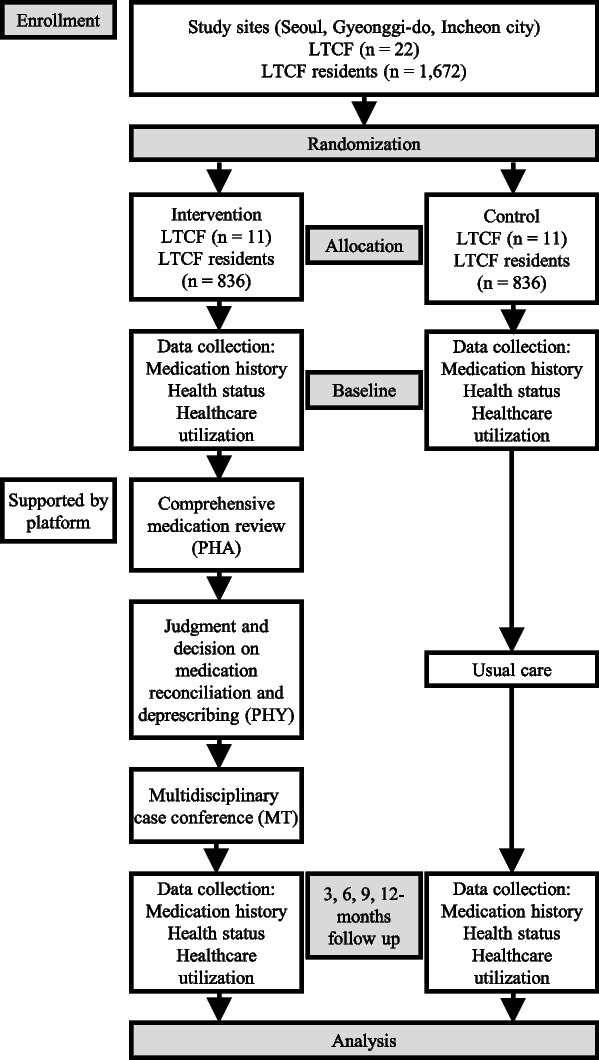
Standard Protocol Items: Recommendations for Interventional Trials (SPIRIT) figure with study timeline and data collection time points. T1 baseline, T2 3 months (end of the intervention), T3 6 months (3 months after the end of the intervention), T4 9 months (6 months after the end of the intervention), T5 12 months (9 months after the end of the intervention), T6 15 months (12 months after the end of the intervention). ADE, adverse drug event; CNS, central nervous system; LTCF, long-term care facility; PIM, potentially inappropriate medication

**Fig. 2:**
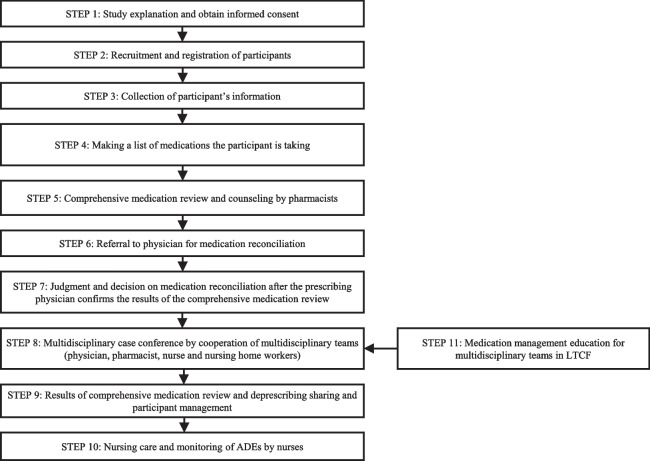
Flow chart with an overview of the study steps. LTCF, long-term care facility; MT, multidisciplinary team; PHA, pharmacist; PHY, physician

This is a prospective, cluster randomized, controlled, and open-label study comparing two LTCF residents’ group care strategies: (1) the intervention group, receiving an MMMP including CMR, deprescribing, and MCC, and (2) the control group, receiving typical usual care. The open procedure is the only possible option because of the nature of the intervention, which requires healthcare workers and participants to not be blinded. The study protocol was designed according to the Consolidated Standard of Reporting Trials (CONSORT) Statement. The Standard Protocol Items: Recommendations for Interventional Trials (SPIRIT) checklist and figure (Fig. 1) were used to prepare the study protocol. A flowchart showing an overview of the study is presented in Fig. 2.

### Recruitment of participants and long-term care facilities

Recruitment for the trial began in April 2022 and is expected to continue until late 2023. The investigators will recruit the LTCFs through direct contact. The nursing home (NH) associations in Seoul, Gyeonggi-do, and Incheon will support recruitment through direct mail to their members, as will the professional association of LTCF physicians. The lead implementation provides methodological support and is responsible for the overall coordination of the study and implementation of interventions.

Participants will be identified through electronic medical records (EMR), history taking, and inter-professional discussions with nurses and research assistants at the LTCFs. Nurses in the LTCF will obtain consent from residents and guardians. If the resident is a patient with dementia and cognitive problems or is unable to sign the consent form, the same will be obtained from the legal representative (spouse, lineal ascendants, or descendants). Recruitment will continue until 76 residents of the LTCF have been included or until 76 residents in one cluster participate.

### Education for pharmacists and nurses in the long-term care facilities

The pharmacist will perform the CMR for the participants, who will be educated on the methodology of performing CMR before the study by attending a course organized by researchers at the college of pharmacy. A separate education session will be organized in each LTCF for nurses and workers, presenting the conduct and documents of the study and introducing them to the platform.

### Participants

To reach the target sample, when a participant is admitted, the nurses and research assistants performing the clinical examination review the inclusion criteria and consider the participant’s eligibility for the study. South Korean is composed of relatively homogeneous ethnic groups.

#### Inclusion and exclusion criteria

The source population will include residents of LTCF. All residents rostered are eligible for participation, provided they fit the inclusion and exclusion criteria.

Participants meeting the following criteria will be eligible for the trial:

- Aged ≥ 65 years
- A resident who meets one or more of the following criteria
  - ` New residents admitted to the LTCF within 4–6 weeks
  - ` Residents discharged within 1 month before the researcher’s visit date
  - ` Those having experienced a fall or fracture within 3 months before the researcher’s visit date.
  - ` Those requiring a long-term care level of 1 (those who were completely dependent on others’ help in daily life)
  - ` Those taking ≥ 10 medications, excluding essential medications
  - ` Those taking medications that may be misused (e.g., narcotic painkillers and inhalers)
  - ` Those taking medications with a narrow margin of safety or high-risk drugs (e.g., narcotic analgesics, antiepileptic drugs, digoxin, theophylline, immunosuppressants, anticancer drugs, anticoagulants, insulin, and benzodiazepines)
  - ` Those with suspected adverse drug events (ADEs) before or during admission to an LTCF
  - ` Those requesting care from visiting physicians, nurses, pharmacists, and caregivers

Participants will be excluded based on the following exclusion criteria:

- ` Those quarantined for infectious diseases
- ` Those receiving regular medication prescriptions at a tertiary general hospital (this is because according to the research team’s analysis, the risk of PIMs is relatively low when the long-term prescription is for more than 90 days)
- ` Those with a predicted life expectancy of less than 6 months by a visiting physiciansor nurse (e.g., terminal cancer)

### Allocation and randomization

LTCF clusters will be randomly allocated in a 1:1 ratio to either the intervention or control group. A randomization sequence will be generated using a computerized system. All participants in one LTCF cluster will be assigned to the same group.

#### Randomization

Computer-generated randomized lists will be drawn before beginning the study. The LTCF clusters will be randomized to either the control group or the intervention group in a 1:1 allocation ratio. An open-label study is the only option because of the involvement of the participants in the medication reconciliation procedure.

#### Blinding

Participants will not be blinded to the allocation because this is not practically or economically feasible. Moreover, given the nature of the intervention, no blinding is possible at the LTCF level or for the researchers, and only the statistician performing the analysis will be blinded; unblinding will occur only after the completion of the analysis.

### Experimental and control interventions

Each participant included in the study will be randomized into one of the following two groups:

- Control group

The control group will receive the typical clinical care, as routinely provided in the LTCFs where the study will take place. The only restriction applied would be that no other planned CMR, deprescribing, or MCC will be performed in the LTCF setting during this phase.

- Intervention group

We developed an MMMP for LTCF residents as a multifaceted intervention that integrates available evidence tools and a team approach within a clinical pathway where decision-making is led by considering participants’ priorities.

The research team including physicians, pharmacists, and nurses will be informed of the approach and program development so the intervention is as “fit for purpose” and as feasible as possible [9]. To foster fidelity, participating visiting physicians, pharmacists, and nurses in LTCF will receive an in-person training session for the program and the entire intervention, as well as a demonstrative manual [9].

This program will be operationalized as a structured clinical pathway in an electronic web-based platform, called “MMMP in LTCF” (pacen-mmmpin.com [PACEN; website.ne.kr]). It serves as a shared record for all visiting physicians, pharmacists, and other relevant workforces participating in the pathway for access and review. It is designed to be used in conjunction with and complement the EMR. This platform records and integrates five domains (Supplementary Text 1).

**Fig. 3:**
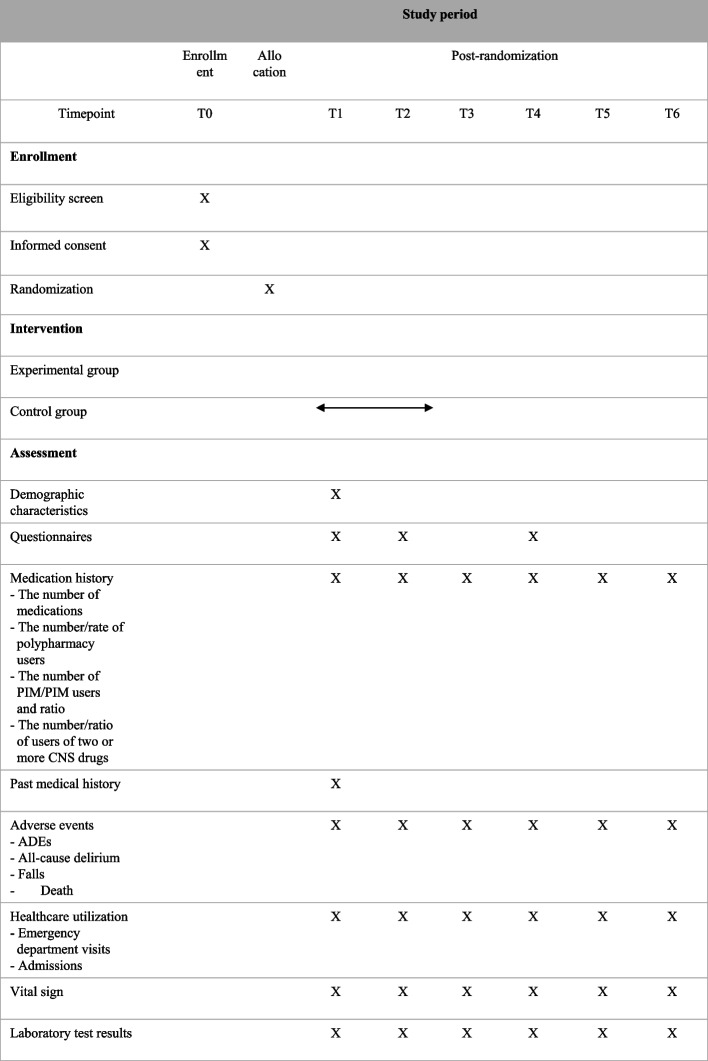
Eleven-operational step-containing multidisciplinary medication management program. ADE, adverse drug event; LTCF, long-term care facility

Operational steps (Fig. 3)

- STEP 1: Explanation of the study and obtaining informed consent
- STEP 2: Recruitment and registration of participants
- STEP 3: Collection of participant’s information
- STEP 4: Listing medications taken by the participant
- STEP 5: CMR and counseling by pharmacists
- STEP 6: Visiting physicians’ referral for medication reconciliation
- STEP 7: Medication reconciliation after the prescribing physician confirms the CMR results.
- STEP 8: Conducting MCC through the cooperation of multidisciplinary teams (visiting physicians, pharmacists, nurses, and NH workers)
- STEP 9: Sharing CMR results, deprescribing, and participant management
- STEP 10: Nursing care and monitoring of ADEs by nurses
- STEP 11: Medication management education for the multidisciplinary teams in the LTCF.

A detailed description of the operational steps is provided in Supplementary Text 2.

### Endpoints/evaluation criteria

The evaluation indices comprise clinical and process evaluation indices, and the clinical evaluation indices comprise primary and secondary outcomes. Primary and secondary outcomes will be measured at baseline (T1), at the end of the intervention (T2), as well as at 3 (T3), 6 (T4), 9 (T5), and 12 months (T6) after the end of the intervention for effectiveness analysis. Figure 1 shows a detailed schedule of data collection.

#### Clinical evaluation indices

##### Primary endpoints

The primary endpoints are (1) ADEs, (2) the number of PIMs and PIM users, (3) the ratio of PIMs and PIM users, (4) the number and ratio of users of two or more central nervous system (CNS) drugs, (5) all-cause delirium, (6) emergency department visits, (7) admissions, and (8) falls at T1-T6.

An ADE is defined as a severe adverse event, i.e., emergency room visits, hospitalizations, falls, and deaths due to “possible” abnormalities according to the World Health Organization-Uppsala Monitoring Centre causality evaluation criteria [10], or adverse drug reactions (ADRs) due to the use of drugs to be wary of or high-risk drugs for older adults. Medication-related information will be recorded to identify and describe the processes of medication change during and after the intervention. Locally acting topical agents without substantial systemic absorption or effects are excluded from the study. Acute short-course medications, such as antibiotics, are also excluded from the counts. This will be ascertained by a pharmacist after medication reconciliation, using dispensing, EMR, and participant information.

##### Secondary endpoints

Secondary endpoints are the number of medications taken and the number and rate of polypharmacy.

#### Process evaluation indices

The participants in the intervention arm of the study will be evaluated. In each cluster of LTCF, the number of CMR cases, requests for medication reconciliation (delivery of CMR results to visiting and external physicians), responses to medication reconciliation, ADE monitoring (medication use-related, such as medication management and ADRs), case management cases, the time spent on CMR and MCC, as well as the experience of guardians, visiting physicians, and nurses in the entire cluster are evaluated at the end of the intervention.

Experience evaluation will provide a full picture of their experience with the intervention and the process of describing it at the initial stages of the intervention, as well as at the end. The guardians of participants, visiting physicians, and nurses in the LTCF will also be asked about their satisfaction with the intervention process and with their care around medications using Likert-type scales, as well as free text responses of the strengths and weaknesses of the process, and whether they would recommend the intervention to a family member or friend.

### Follow-up and data collection

The evaluation will be performed at six different time points: T1–T6. The study procedure and data collection are detailed in Fig. 1 and have been established as per the SPIRIT guidelines. A research assistant will collect data from the pharmacists in the intervention group. For the control group, they will visit the LTCF and obtain baseline data from the institutional documents or EMR about the participant’s characteristics, including age, sex, general condition (long-term care level, disability, behavioral status [whether bedridden], and bedsores, if any), medical history (comorbidity), family history, vital signs, and laboratory test results. In addition, medication history, medication reconciliation, and outcomes are recorded as part of the clinical intervention. Data for security outcomes (falls, hospitalization, emergency department visits, and death) will be collected from the records or EMR directly from participating LTCFs. In the case of hospitalization or death of a participant during the follow-up period, no questionnaire for subsequent outcomes will be filled.

### Data protection

Researchers (physicians, pharmacists, nurses, and other associates) and multidisciplinary teams in LTCF will access the platform using their unique identifications. A multidisciplinary team in an LTCF can manage and view only the information on the LTCF participants to which it belongs.

### Statistical considerations

#### Sample size, power, and statistical methods

Sample size calculations for the cluster RCT were performed by the team biostatistician. The required number of participants was calculated from the primary endpoint, that is, the number of PIM users. Based on recent literature, PIM users in LTCF comprised 40.7% [6], and the expected effect difference through literature review was an odds ratio of 0.41 [11]. Calculating the sample size using Sample Size Calculator V2.0 (Health Services Research Unit, University of Aberdeen) with a two-sided significance level of 5% and 95% power, 1,430 participants are needed (715 in each group). A total of 1,672 participants (836 in each group) should be included to prevent a dropout rate of 15% (discharge, hospitalization, death, etc.).

#### Data analysis

The analysis will follow the intention-to-treat approach.

All analyses will be performed using SAS software (version 9.4; Cary, NC, USA). Statistical significance is defined as *p* < 0.05. This methodology follows the Consolidated Standards of Reporting Trials Statement (http://www.consort-statement.org/consort-statement) [12]. Interim analysis is planned at midpoint after the start of participants’ enrollment and when issues arise during the study, and missing data will not be replaced. If issues arise during the study, the study design will be modified.

#### Data management and data quality

Quality assurance and control of data will be conducted in accordance with Good Clinical Practice (decisions of November 24, 2006) [13].

Participant identifiers in electronic files and paper documents will be kept strictly confidential. Electronic and paper documents will be stored for 10 years [13].

### Loss to follow-up

We anticipated a conservative maximum loss to follow-up rate of 15%. Participants lost to follow-up will be classified as such because they elected to no longer be part of any further follow-up (withdrawal of consent) or were unable to participate because of discharge, hospitalization, death, etc. In the case of discontinuation of research at an LTCF before the completion of the registration of 76 participants, the target of one cluster, participants already registered at the LTCF are included in the study for intervention and follow-up, and the remaining participants are recruited from other similarly-sized LTCFs (number of residents). In cases where participants who are lost to follow-up do not explicitly withdraw consent, medication and healthcare utilization data will still be collected from participant charts via chart audits where possible [9].

### Safety and monitoring

Details of any clinically adverse symptoms or effects at the research data collection points detected by a nurse or NH worker and reported to the researcher will be acted upon immediately and at the same time will be securely provided to their visiting physicians and pharmacist at that time. If a participant experiences a serious adverse event (SAE), the study team will notify the research ethics board of any SAE thought to be related to the study, using the standard SAE form.

## Discussion

This study protocol describes a pragmatic, cluster RCT designed to assess the effect of an MMMP in an LTCF with well-established inter-professional collaborations to ensure a reduction in polypharmacy and PIM use.

In the past decade, numerous studies have anchored the CMR and deprescribing as a safe and powerful tool to enhance clinical outcomes for older patients and NH residents [14], and have been shown to be beneficial for relevant clinical outcomes [11, 14]. However, the evidence for the effect of interventions to reduce polypharmacy and PIM use on health outcomes in LTCF settings is inconsistent and difficult to implement [9]. A study in an LTCF targeting deprescribing of anticholinergic and sedative medicines showed that medication reduction resulted in reductions in psychotropic drug side effects, falls, and depression and frailty scores [15]. However, another systematic review reported that no firm conclusions on the effects could be drawn [16]. Moreover, several reviews recommended further RCTs evaluating multidisciplinary interventions and clinical outcomes in specific high-risk populations, such as LTCF populations.

To the best of our knowledge, the impact of CMR and deprescribing has not been clarified in previous research, and it has not been determined whether negative health outcomes are reversible if polypharmacy and PIM use are reduced. In this respect, our study is novel and unique in its design, by introducing an operationalized structured clinical pathway aimed at tackling polypharmacy and PIM use in LTCF settings while simultaneously considering participants’ goals and priorities for treatment through the participation of participants and guardians. In addition, several explicit tools and deprescription guidelines are available on the platform to check medications that may be inappropriate or suitable for deprescription among LTCF residents. Furthermore, most of the ongoing studies are limited to the Americas and Europe; therefore, it is time to develop and implement programs suitable for Asian conditions, and it is necessary to confirm the clinical effects of comprehensive and systematic medication management in LTCFs through the operation of developed programs. We will also evaluate participants, guardians, and physicians’ experiences of participating in the program.

In our study, participants and researchers are not blinded due to the nature of the intervention (open-label study). To minimize the bias caused by this, we implemented cluster study design. Additionally, in order to minimize performance and detection bias, the study will be conducted according to a pre-determined method according to the protocol. Moreover, if there are indicators with significant differences between the intervention group and the control group when analyzing the results later, they will be adjusted using statistical techniques. Furthermore, in order to minimize attrition bias in the outcome analysis, we will follow the intention-to-treat approach, and data on those who dropped out will be obtained and analyzed from data of the National Health Insurance Sharing Service (It refers to a vast amount of data amounting to 1.3 trillion cases, including demographic information about each patient, details of prescribed drugs, prescribed medical tests and health checkup results, and long-term care insurance data for the elderly, of all citizens).

We developed a multidisciplinary medication management program (comprehensive medication reviews, deprescribing, and multidisciplinary case conferences) to assess its impact on medication use and health problems among LTCF residents. This program, with a high level of evidence as a study with a long-term follow-up period targeting a large population, is immediately scalable, and the results of the trial, if successful, are anticipated to be directly translatable into clinical practice [9] and will become a part of routine preventive care in LTCF residents. Evidence from this study will help to understand the provision of a systematic approach to reducing the burden of polypharmacy and PIMs, as well as providing a systematic clinical pathway for implementation. Based on our results, intervention methods suitable for other settings should be developed.

### Supplementary Information

**Supplementary Material 1.**

## Data Availability

Not applicable.

## Abbreviations

ADE: Adverse drug event
ADR: Adverse drug reaction
CMR: Comprehensive medication review
CNS: Central nervous system
EMR: Electronic medical record
LTCF: Long-term care facility
MCC: Multidisciplinary case conference
MMMP: Multidisciplinary medication management program
NH: Nursing home
PIM: Polypharmacy and potentially inappropriate medication
RCT: Randomized controlled trial
SAE: Serious adverse event
