# Supplementary material for: Development of a multidisciplinary medication management program in nursing homes: protocol for a randomized controlled trial: Multidisciplinary medication management in nursing homes
Source: BMC Geriatr. 2024 Mar 4;24:218. doi: 10.1186/s12877-024-04844-2 (PMC10910765; doi:10.1186/s12877-024-04844-2)
Supplement: Supplementary file 1 — Supplementary Material 1. [file 12877_2024_4844_MOESM1_ESM.docx]

**Development of a multidisciplinary medication management program in nursing homes: Protocol for a randomized controlled trial**

Subtitle: Multidisciplinary medication management in nursing homes

**Supplementary Text 1. Detailed description of each domain of the platform for multidisciplinary medication management program in long-term care facilities**

1. Domain 1. Participant characteristics: this domain covers participants’ ID, name, age, sex, visiting number, and name of the long-term care facility (LTCF).

2. Domain 2. Comprehensive medication review: Target medications include all prescribed, over-the-counter, and functional health foods, and classification is made according to whether the applicable medications are taken regularly or when needed; for each medication, the brand name, ATC code, dosage form, dose, number of doses per day, and regularity of taking (if taking as needed, number of doses in the past week), the start and end dates of administration, and the name of the prescribed medical institution are entered. Medication formulations include tablets/capsules, extended-release/enteric-coated tablets (capsules), sublingual tablets, powders, syrups, suppositories, injections, patches, eye drops, eye ointments, ointments/creams, inhalers, and nasal sprays. For medications to be taken when necessary, research assistants can choose from non-steroidal anti-inflammatory drugs (NSAIDs), non-NSAID analgesics, benzodiazepines, sleeping pills other than benzodiazepines, narcotics, antihistamines, antispasmodics, antacids, laxatives, antidiarrheals, insulin, and nitroglycerin. For over-the-counter medicines and functional health foods, the research assistants will be able to select from the drug name, red ginseng, calcium supplement, multivitamin, omega-3, aspirin, general cold medicine, anti-inflammatory painkillers, Ginkgo leaf preparations, and other types, and enter the number of doses per day and the reason for taking them. Taken together, the number of medications taken regularly, number of prescription medications, over-the-counter medicines, functional health foods, number of medications taken when necessary (taken at least once in the past week), and the presence of polypharmacy are checked. These stored medications will also be integrated into an automatic electronic screen to flag PIMs or the cumulative medication burden in older adults. Medication treatment, such as confirmation of indication (identification of unnecessary drugs), overlapping administration of the same ingredient/similar efficacy group, confirmation of drug effect, drug-drug interaction, drug-disease interaction (contraindications), and drug-food interaction is reviewed, and dose adequacy, dosage form selection, adverse drug reactions, medications to be cautious of by older adults, medication compliance, suitability for use, and the requirement of additional drug treatment are evaluated. Finally, through a review, medications that require intervention are determined, and the reasons for intervention, contents of intervention (discontinuation, change, monitoring, participant/caregiver education, referral, etc.), and intervention plans will be provided to support tapering and/or deprescribing.

3. Domain 3 Intervention: The name of the pharmacist who performed the comprehensive medication review, results of the comprehensive medication review, and target medication for adjustment are entered. Through a comprehensive medication review, the following medications are identified: inappropriate medications that should be prioritized for management in older adults in LTCFs; those considered as high-risk medications for older adults in LTCFs; those meeting the Beer’s criteria 2019; those meeting the Screening Tool of Older Persons' Prescriptions (STOPP) and Screening Tool to Alert to Right Treatment (START) criteria version 2, 2015; and anticholinergic medications with a high anticholinergic burden.

4. Domain 4. Outcomes: The process evaluation index is the number of comprehensive medication review cases for residents in the entire institution at the LTCF institutional level, number of medication reconciliation requests (delivery of drug review results to internal and external prescribing doctors), number of medication reconciliation responses, monitoring of medication abnormalities (medication management in cases related to drug use, such as adverse drug reactions), number of multidisciplinary case management conferences, time spent on medication review, time spent on case management conferences, and experience evaluation of guardians, physicians, and nurses. The clinical evaluation index consists of primary and secondary endpoints, and the results of each LTCF and each participant are all entered at the end of the intervention and at 3, 6, 9, and 12 months after the end compared to the baseline value. The primary endpoints are adverse drug events, number and ratio of PIMs and PIM users, number and ratio of users of two or more central nervous system drugs, all-cause delirium, emergency department visits, admissions, and falls. Secondary endpoints are the number of medications taken, and the number and rate of polypharmacy use.

5. Domain 5. Appendix: These include various forms and reference materials used for comprehensive medication reviews The forms include explanations and consent forms for participation, medication reconciliation request forms, medication reconciliation decision and response forms, multidisciplinary case conference forms, nursing care forms, medication intake forms, and participant safety (accident) report forms. The reference materials include a list of the following: inappropriate medications that should be managed as a priority for older adults in LTCFs; medications to be cautious of by older adults in LTCFs; high-risk medications for older adults in LTCFs; medications meeting the Beer's criteria, 2019; medications meeting the STOPP and START criteria version 2, 2015; anticholinergic burden score of anticholinergic medications; ICD-10 2019 code; guidelines for the safe use of hypnotic sedatives used frequently; guidelines for safe medication use; and clinical guidelines for the behavioral and psychological symptoms of dementia.

**Development of a multidisciplinary medication management program in nursing homes: Protocol for a randomized controlled trial**

Subtitle: Multidisciplinary medication management in nursing homes

**Supplementary Text 2. Step-by-step detailed description of the operational steps**

STEP 1: Explanation of the study and obtaining informed consent

A nurse in the long-term care facility (LTCF) explains the research using promotional brochures or videos to participants who meet the inclusion criteria and their guardians and obtains informed consent. If a participant has dementia or is unable to sign the consent form, consent is obtained only from the guardian (the legal representative).

STEP 2: Recruitment and registration of participants

A research assistant registers participants who meet the inclusion/exclusion criteria described above.

STEP 3: Collection of participant’s information

Based on the existing format of LTCF, any available data on the participants’ characteristics relevant to medications, demographic characteristics, and anthropometric data are collected. This will be performed by a research assistant, along with the collection of baseline outcome data.

STEP 4: Listing medications taken by the participant

Our study will acquire data through a collaboration between the National Health Insurance Service (NHIS) and the Health Insurance Review and Assessment Service (HIRA). Using the records, including the medication dispensing records of the LTCF, NHIS, and HIRA, acquired information such as medication name, medication code, dosage, dose per serving, number of doses, number of days of administration, prescribed medication information, diagnosis, and name of the prescribing medical institution will be entered into the platform. Based on these data, it is possible to formalize a complete list of medications taken by the participants.

STEP 5: Comprehensive medication review and counseling by pharmacists

A comprehensive medication review consists of a pharmaceutical analysis of prescriptions. A comprehensive medication review will be conducted on the participants’ medications, including the accuracy of dose and frequency, appropriateness, and indications. This review also focuses on medications suitable for discontinuation or dose reduction, supported by the reference materials of the platform and evidence resources for deprescribing, reported medication side effects by the participants, and a review of the participants’ medication experience. Using reference materials within the platform, overlapping medications, interactions, and adverse drug events (ADEs; side effects) will be checked, the results recorded on the platform, and the results of medication use assessments documented (creation of a comprehensive medication review form and medication reconciliation request form).

The results of the review will be structured as propositions for medication regimen modifications tailored to the clinical situation of each participant. These propositions include continuation, discontinuation, regimen modifications (administration form, time, dose, or frequency), withdrawal or tapering of non-beneficial medications (deprescribing), switching to safer medications, and introduction of new medications in case of prescription omission.

Comprehensive medication review and medication reconciliation request forms prepared by the pharmacist are delivered to the physician. Conversely, if a physician determines that a comprehensive medication review is necessary, they can request a review by a pharmacist.

STEP 6: Physician referral for medication reconciliation

If the pharmacist determines that medication reconciliation is necessary as a result of a comprehensive medication review, he or she requests medication reconciliation from the prescribing physician (delivery of the comprehensive medication review form and medication reconciliation request form).

STEP 7: Medication reconciliation after the prescribing physician confirms the results of a comprehensive medication review.

When medication is prescribed by an internal physician in an LTCF, the physician reviews the pharmacist's comprehensive medication review and fills out the medication reconciliation decision and the response form. When medication is prescribed by an external physician outside the LTCF, the medication reconciliation request form is delivered in writing through the guardian, and the prescribing physician who confirms that action has been taken, prepares the medication reconciliation decision and response form, and delivers it to the LTCF through the guardian.

Actions are classified as no medication reconciliation, medication reconciliation present (discontinuation of medication, change of prescription to another medication, adjustment of prescription dose, route of administration, change of dosage form, addition of prescription medication, etc.), and medication reconciliation required but no reconciliation.

STEP 8: Conducting multidisciplinary case conference through the cooperation of multidisciplinary teams (physicians, pharmacists, nurses, and nursing home workers)

Propositions by pharmacists will be discussed with the physicians, nurses, and nursing home workers with the goal of developing a deprescribing plan that will be validated with the participants or their guardians before being enacted.

Multidisciplinary teams (physicians, pharmacists, nurses, and nursing home workers) attend a multidisciplinary case conference once a month to discuss with participants who need intervention, identify problems, establish an intervention plan, and then conduct medication reconciliation for participants who need intervention through multidisciplinary discussions.

After the conference, the pharmacist creates a multidisciplinary case conference form.

STEP 9: Sharing of comprehensive medication review results, deprescribing, and participant management

In the case of outpatient treatment at an external hospital or clinic (when medication is prescribed by an external physician outside the LTCF), the results of the comprehensive medication review and deprescription are delivered in writing through the guardian.

STEP 10: Nursing care and monitoring of ADEs by nurses

Nursing care includes monitoring basic vital signs, recording possible ADEs that may have occurred before or during admission to an LTCF, and identifying adverse events. In addition, a medication intake form is filled out, and participant accidents are monitored and reported.

STEP 11: Medication management education for the multidisciplinary teams in the LTCF

The educational content includes the importance of proper management of multidisciplinary medication use, program information, examples of appropriate management of multidisciplinary medication use, and actual medication reconciliation. Education is provided in person or through virtual or video consultations.
